# Recommendations for myeloid-derived suppressor cell nomenclature and characterization standards

**DOI:** 10.1038/ncomms12150

**Published:** 2016-07-06

**Authors:** Vincenzo Bronte, Sven Brandau, Shu-Hsia Chen, Mario P. Colombo, Alan B. Frey, Tim F. Greten, Susanna Mandruzzato, Peter J. Murray, Augusto Ochoa, Suzanne Ostrand-Rosenberg, Paulo C. Rodriguez, Antonio Sica, Viktor Umansky, Robert H. Vonderheide, Dmitry I. Gabrilovich

**Affiliations:** 1 Department of Medicine, University Hospital, University of Verona, Verona 37134, Italy; 2 Department of Otorhinolaryngology, University Hospital Essen, Essen D-45122, Germany; 3 Department of Oncological Sciences, Tisch Cancer Institute, Immunology Institute, Icahn School of Medicine at Mount Sinai, New York, New York 10029, USA; 4 Department of Experimental Oncology and Molecular Medicine, Molecular Immunology Unit, Fondazione IRCCS Istituto Nazionale dei Tumori, Milano 20133, Italy; 5 New York University School of Medicine, New York, New York 10029, USA; 6 GI-Malignancy Section, Thoracic and GI Oncology Branch, NCI, Bethesda, Maryland 20892, USA; 7 Department of Surgery, Oncology and Gastroenterology, Section of Oncology and Immunology, University of Padova, Padova 35128, Italy; 8 Veneto Institute of Oncology IOV-IRCCS, Padova 35128, Italy; 9 Departments of Infectious Diseases and Immunology, St Jude Children's Research Hospital, Memphis, Tennessee 38105, USA; 10 Stanley S. Scott Cancer Center, Louisiana State University, New Orleans, Louisiana 70112, USA; 11 University of Maryland Baltimore County, Baltimore, Maryland 21250, USA; 12 Georgia Regents University Cancer Center, Augusta, Georgia 30912, USA; 13 Humanitas Clinical and Research Center, Via Manzoni 56, Rozzano, Milan 20089, Italy; 14 Department of Pharmaceutical Sciences, Università del Piemonte Orientale ‘Amedeo Avogadro', via Bovio 6, Novara 20089, Italy; 15 Skin Cancer Unit, German Cancer Research Center (DKFZ), Heidelberg 69120, Germany; 16 Department of Dermatology, Venereology and Allergology, University Medical Center Mannheim, Ruprecht-Karl University of Heidelberg, Mannheim 69120, Germany; 17 Abramson Cancer Center, University of Pennsylvania School of Medicine, Philadelphia, Pennsylvania 19104, USA; 18 Translational Tumor Immunology, The Wistar Institute, Philadelphia, Pennsylvania 19104, USA

## Abstract

Myeloid-derived suppressor cells (MDSCs) have emerged as major regulators of immune responses in cancer and other pathological conditions. In recent years, ample evidence supports key contributions of MDSC to tumour progression through both immune-mediated mechanisms and those not directly associated with immune suppression. MDSC are the subject of intensive research with >500 papers published in 2015 alone. However, the phenotypic, morphological and functional heterogeneity of these cells generates confusion in investigation and analysis of their roles in inflammatory responses. The purpose of this communication is to suggest characterization standards in the burgeoning field of MDSC research.

## Abstract (web summary)

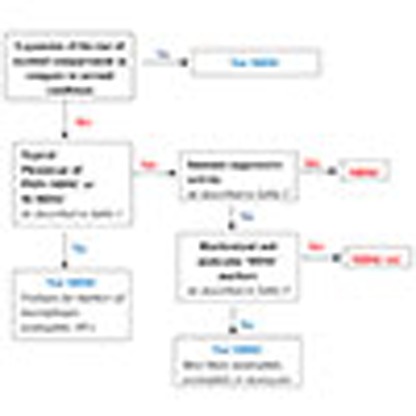
Myeloid-derived suppressor cells (MDSC) are a heterogeneous population expanded in cancer and other chronic inflammatory conditions. Here the authors identify the challenges and propose a set of minimal reporting guidelines for mouse and human MDSC.

## Definition of MDSC

At steady-state, myelopoiesis is a structured process where progeny of common precursors acquire specific markers and functions of circulating leucocytes, and in doing so progressively lose the ability to self-renew. The turnover of mature leucocytes is substantial, with billions of cells generated and replaced daily. A variety of pathological conditions can perturb the steady supply of leucocytes resulting in emergency myelopoiesis^1^, which serves to provide cells to eliminate potential threats, either abnormal cellular growth (cancer), infectious agents or tissue damage. If these conditions resolve quickly, the balance of myeloid cells is restored without negative consequences for the host. However, a number of conditions associated with various types of chronic inflammation, autoimmune diseases and cancer results in aberrant, sustained myelopoiesis characterized by the accumulation of immature myeloid cells that deviate from the standard path of differentiation. These cells are distinct from mature, terminally differentiated myeloid cells (macrophage, dendritic cells or neutrophils) and have an activation programme (pathologic activation), which is different from that of mature myeloid cells.

Early studies on inflammation in the mouse highlighted a shared, systemic expansion of myeloid cells bearing the markers CD11b (CR3A or integrin αM) and Gr-1 (anti-Gr-1 mAbs recognize epitopes common to Ly6C and Ly6G) (refs 2, 3, 4, 5). It became apparent that CD11b^+^Gr-1^+^ cells were heterogeneous, which generated uncertainty in description of these cells among different groups of investigators, an ambiguity amplified by the use of different acronyms to define these cells. In an attempt to codify analysis of the nature and clinical significance of these cells, 9 years ago a group of investigators suggested to utilize the term of ‘myeloid-derived suppressor cells' (MDSC), informed by the myeloid origin, the immune-suppressive function and the systemic expansion of MDSC in a cancer-related context^6^. The initial intent to introduce MDSC nomenclature was not to define a novel population of myeloid cells (at that point being clear that MDSC are not a distinct myeloid lineage), but to provide a term that captured the function, origin and heterogeneity of the cells and offered a framework to guide studies on these remarkable players in tumour-dependent immune dysfunction.

Since the inception of this term, interest in MDSC has blossomed. MDSCs are implicated in various aspects of immune regulation in diseases that involve chronic inflammation, especially cancer, but also infection, autoimmune diseases, trauma, graft versus host disease and so on. Recently, evidence of the clinical significance of MDSC in cancer has emerged. Therefore, despite realization that the term ‘MDSC' may not be optimal, we feel that it is purposeful and should be retained to assure consistency as the field continues to develop.

However, several notions about MDSC now require reassessment. First, the cellular nature of MDSC is now better defined and includes two major subsets based on their phenotypic and morphological features: polymorphonuclear (PMN) and monocytic (M)-MDSC, and to reflect those discoveries, the terms PMN-MDSC and M-MDSC were introduced^7^,^8^. Initially we and others used the term ‘granulocytic MDSC' to describe PMN-MDSC, but now we believe that the latter term better defines this MDSC subset, since PMN-MDSC are phenotypically distinct from steady-state neutrophils (having less granules, altered buoyancy, reduced CD16 and CD62L, and increased arginase 1, peroxynitrite, CD11b and CD66b) (refs 9, 10). It is apparent now that PMN-MDSC and M-MDSC are not only phenotypically and morphologically distinct, but also have unique (although partially overlapping) functional characteristics and biochemical traits, which reflect their different roles under various pathological conditions. Therefore, we believe that the definition of MDSC in scientific reporting needs to include the specific subset of MDSCs under investigation.

Second, even though functional analyses of immunoregulatory activity are often lacking for practical reasons (typically relating to the paucity of MDSC in human samples), the systemic expansion of circulating myeloid cells and the correlation with clinical outcomes have been reported for both solid and hematologic human malignancies, confirming the concept that tumours can influence, at different stages, the ‘distant' hematopoietic compartment^11^,^12^. The recent identification of specialized molecular programs that orchestrate MDSC differentiation, joined with high-throughput technologies (see below), has provided new insight in the multifaceted and unique myeloid cell development leading to MDSC generation.

The marked growth of publications over the last years has led to deviation from the original intent of the MDSC nomenclature. The objective of this review article is not to provide a comprehensive analysis of the MDSC biology, which has been done in many recent publications, but to suggest minimal phenotypic, molecular and functional criteria for reporting and classifying MDSCs. We also propose to integrate the increasing observations, in which expansion of cells with MDSC morphology, phenotype and main biochemical features is not accompanied by immune suppression. The criteria for defining cells as MDSCs should include phenotypic and functional, and, if possible, molecular characteristics described below.

## Phenotypic markers to define MDSC

**Figure 1:**
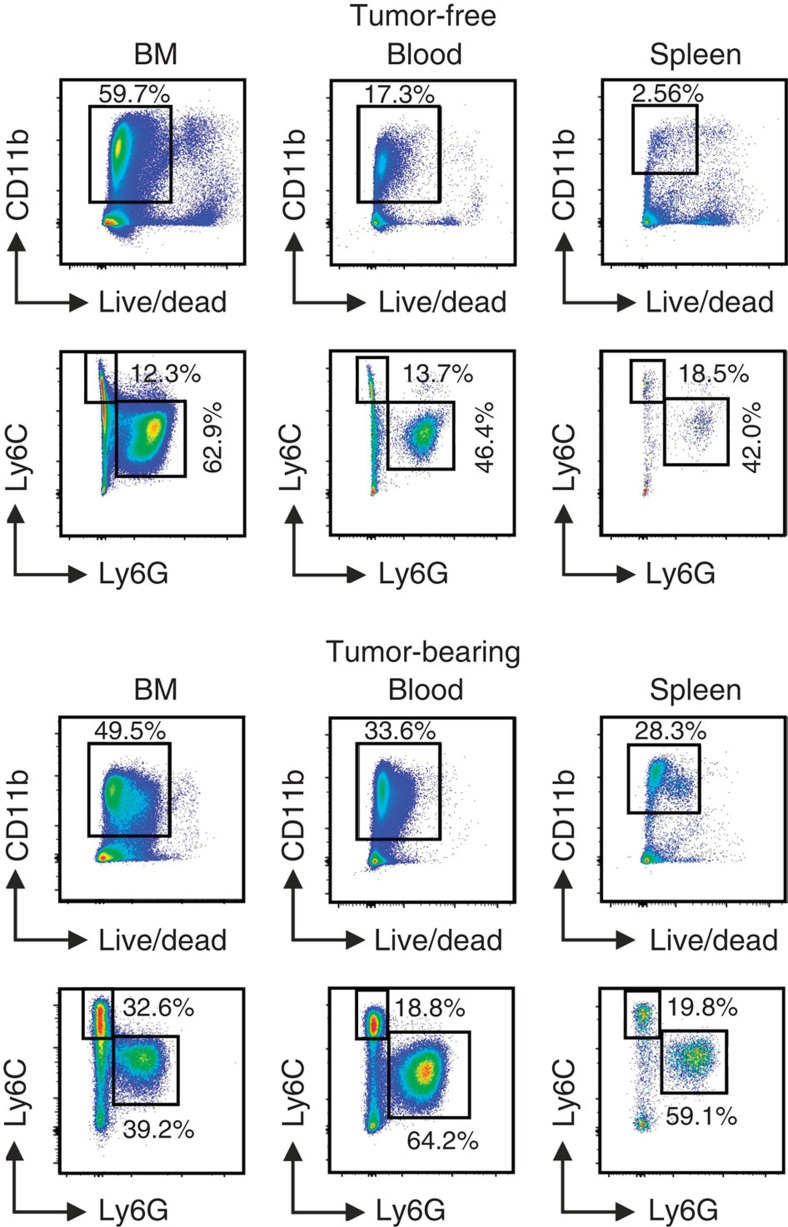
Gating strategy for the identification of mouse MDSC subsets. Gating strategy used to define MDSC subpopulations in BM, blood and spleen of C57Bl/6 tumour-free or MCA203 tumour-bearing mice. After exclusion of doublets (not shown), live CD11b^+^ cells were gated and the proportion of Ly6C and Ly6G cells was evaluated.

In mice, MDSCs historically were defined as cells expressing both Gr-1 and CD11b markers. Although initially useful in identifying MDSC, the use of this original criterion is no longer sufficient since subpopulations have been shown to exist: PMN-MDSC (CD11b^+^Ly6G^+^Ly6C^lo^) and M-MDSC (CD11b^+^Ly6G^−^Ly6C^hi^). Example of staining is provided in Fig. 1. These cell-surface markers provide only an initial framework for characterization and can be complemented by various other markers (described in detail elsewhere)^13^,^14^.

**Figure 2:**
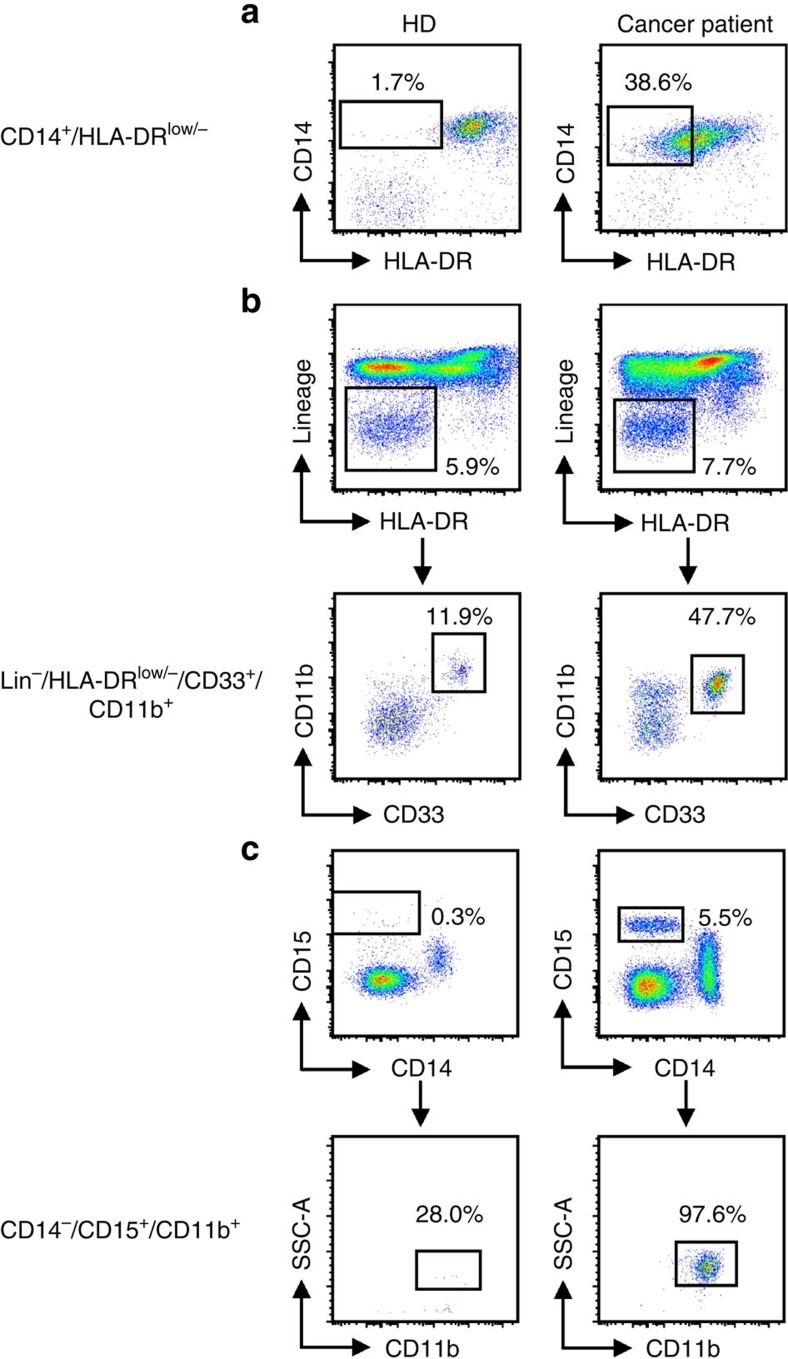
Gating strategy for the identification of MDSC subsets in the peripheral blood of healthy donors and melanoma patients. Doublets were excluded and live PBMC were gated (not shown). (**a**) CD14^+^HLA-DR^−/lo^ M-MDSC. Monocytes were gated on the basis of FSC and SSC parameters and HLA-DR downregulation was defined by FMO control. (**b**) Lin^−^HLA-DR^−^CD33^+^ eMDSC. (**c**) CD14^−^CD15^+^CD11b^+^ PMN-MDSC.

In human peripheral blood mononuclear cell (PBMC), the equivalent to PMN-MDSC are defined as CD11b^+^CD14^−^CD15^+^ or CD11b^+^CD14^−^CD66b^+^ and M-MDSC as CD11b^+^CD14^+^HLA-DR^−/lo^CD15^−^. CD33 myeloid marker can be used instead of CD11b since very few CD15^+^ cells are CD11b^−^. While M-MDSC express the myeloid marker CD33, PMN-MDSC display CD33^dim^ staining^15^. Lin^−^ (including CD3, CD14, CD15, CD19, CD56) HLA-DR^−^CD33^+^ cells contain mixed groups of MDSC comprising more immature progenitors. These cells have been defined as immature MDSC and the mouse equivalent is yet to be identified. However, since some immaturity is a common trait of all MDSC subsets, we propose to define them more properly as ‘early-stage MDSC' (eMDSC). At this point, it appears that any characterization of MDSC needs to include each of these cell populations (Table 1). Example of staining is provided in Fig. 2. A recent report based on the experience of many laboratories around the world, suggested specific gating parameters for harmonized phenotyping of human MDSC by flow cytometry^16^. Although various additional populations of myeloid cells were described in that analysis, their morphological, biochemical or functional distinctions from each other and from PMN-MDSC, M-MDSC or eMDSC are not yet clear. In contrast to the three main populations, which are found (at various frequencies) in all types of cancer and every pathological condition, some of those cells may exist in one type of cancer but absent in the other. It is likely that there is a substantial overlap between cells belonging to different subsets. Therefore, more detailed analysis of MDSC populations is needed for better characterization. However, the minimum (and currently sufficient) requirements for definition and characterization of human MDSCs (PMN-MDSCs, M-MDSCs or eMDSCs) according to the phenotypic criteria are described in Table 1. Detailed discussion of the specific gating criteria for phenotyping of mouse and human MDSC can be also found elsewhere^17^. These gating criteria cannot discriminate monocytes from M-MDSCs and neutrophils from PMN-MDSC since at present there are no combinations of markers unique to MDSC. Presently, the only method allowing for separation of neutrophils from PMN-MDSC is gradient centrifugation using 1.077 g l^−1^ density (standard Ficoll gradient used for the isolation of mononuclear cells). PMN-MDSC are enriched in low density (mononuclear cell fraction), whereas neutrophils are high-density cells^15^. The limitations of this approach are unavoidable. Low-density fraction contains not only PMN-MDSC, but some activated neutrophils and probably some PMN-MDSC can pass through gradient and contaminate high-density fraction of neutrophils. Therefore, CD11b^+^CD14^−^CD15^+^/CD66b^+^ cells in low-density fraction contain both PMN-MDSC and neutrophils. These cells are heterogeneous in their morphology (containing both mature and immature cells)^18^. Since functional, biochemical and genomic characterization is performed on entire population of cells, and single-cell functional analysis is not yet feasible, the precise nature of PMN-MDSC remains unclear. An important goal for future studies is to define cell-surface markers and gating strategies that uniquely identify the different populations of MDSC. One of the most pressing unmet issues is to determine markers of MDSC that would allow detection of cells in unseparated peripheral blood.

It is important to emphasize that the method for collecting and analysing MDSC can influence the results: freezing of samples lead to substantial loss of cells, especially PMN-MDSC^19^, and thus comparison with freshly isolated cells is necessary.

Furthermore, one of the main challenges in the field is the phenotypic characterization of MDSC in tissues by immunohistochemistry without isolation and functional characterization of the cells. In mice, Gr-1 or Ly6G staining is used to identify tumour-associated MDSC. However, the distinction between neutrophils or monocytes and MDSC in frozen or paraffin-embedded tissues is impossible. In human samples similar confusion of MDSC with monocytes and neutrophils exists. Similarly, CD33, a myeloid marker that sometimes is used for identification of MDSC, does not allow for distinction of MDSC from macrophages, dendritic cells or other myeloid cells in tissues. Recently, a combination of CD33 with S100A9 was suggested to identify MDSCs^20^. However, although this pair of markers excludes most dendritic cells and macrophages (due to their low expression of S100A9), it does not permit a clear distinction of MDSC from neutrophils. Thus, at this moment, clear phenotypic characterization of human and mouse MDSC by immunohistochemistry is lacking.

It is important to emphasize that phenotypic evaluation is a starting point for the analysis of MDSC (see the proposed algorithm below) since it defines population of cells in both naive and pathologically changed hosts. However, expansion of myeloid compartment under pathologic conditions is a critical, first step that allow for further evaluation of MDSC. For instance, in healthy donor cells with typical PMN-MDSC phenotype in the peripheral blood mononuclear cell fraction are practically undetectable and cells with M-MDSC phenotype are present in much smaller numbers than in patients with either melanoma or chronic infections^11^. In mice, similar situation exists in spleen. The proportion of splenic MDSCs increases from 1–2% in naive mice to 10–15% or more in many cancers. In bone marrow and peripheral blood of mice the differences in the proportion of the cells are smaller (usually increase is <twofold) (refs 8, 21).

## Functional assays to define MDSC

The ability to suppress immune cells is an important characteristic of MDSC. Although MDSC were implicated in suppression of different cells of the immune system such as NK and B cells^22^,^23^,^24^, inhibition of T cells is the ‘gold' standard for evaluation of MDSC function and inhibition of T-cell activity appears to be sufficient for designation of cells as MDSC, provided that they meet the phenotypic criteria described above. Different methods for the evaluation of T-cell suppression are used in the literature (summarized in Table 2) and can be divided into two groups. One method assesses antigen-specific suppression and utilizes antigen-specific T cells, activated with cognate peptides or in an allogeneic mixed leucocyte reaction (MLR), in the presence of titrated numbers of purified input MDSC. The second, non-specific suppression, uses T cells activated by CD3 antibody, alone or in combination with CD28 (immobilized or presented by APC), or with lectins (ConA in mice or PHA in humans, although an influence of lectins on other cell types contained in the *in vitro* assay is a concern for potential technical biases). However, antigen-specific suppression assays are preferable when possible, since these assays are likely more relevant to *in vivo* conditions. Various methods have been used to determine T-cell function activity modulated by MDSC. Measurement of T-cell proliferation (either with ^3^H-thymidine incorporation or by CFSE dilution) or inhibition of interferon (IFN)-γ production (ELISPOT or intracellular staining) provides experimental evidence supporting functions associated with MDSC. It is important to note that, under some experimental conditions, MDSC could cause inhibition of T-cell proliferation without affecting IFN-γ production and vice versa. The titration of input MDSC numbers in these tests (and the cognate loss of suppression as the cells are titrated down) is a critical experimental tool to help eliminating the possibility of artefacts due to MDSC viability and to aid in comparison between experiments. One of the critical issues to consider is the use of appropriate controls—cells with the same phenotype and preferably from the same tissues from either healthy donors or naive mice. It is important to point out that there are several exceptions since neutrophils (cells with the phenotype similar to PMN-MDSC) are rarely present in the PBMC fraction of healthy donors. In contrast to PMN-MDSC from tumour-bearing mice and cancer patients, neutrophils are largely absent from lymph nodes of naive mice and healthy donors.

The evaluation of tolerogenic activity of MDSC *in vivo* by using adoptive transfer of MDSC and antigen-specific T cells to syngeneic recipients, with subsequent stimulation of antigen-specific T cells with cognate peptide, provides the most comprehensive way to study different pathways of MDSC activity. However, this method requires considerable effort and probably is not feasible for routine evaluation of MDSC activity. Examples of the *in vivo* tolerogenic use of MDSCs could be found in several publications^25^,^26^.

In humans, evaluation of MDSC effects on antigen-specific immune responses is difficult due to the limitation in generation of antigen-specific T cells. Recent advances allow for transduction of human T cells with lentiviruses expressing cloned T-cell receptors, which permits analysis of suppression in antigen-specific assays using cognate peptides in the presence of autologous antigen-presenting cells and MDSC. Currently, a three-way allogeneic MLR is a reliable method for functional assessment of human MDSC. This assay utilizes cells obtained from a pair of unrelated healthy donors: one is the source of T cells, the other one provides APCs. The pair is selected based on strong T-cell proliferative or IFN-γ responses of the responder, and aliquots of cells can be stored for use in subsequent iterative experiments. MDSCs from cancer patients are tested in MLR at different ratios compared with responder T cells. The assay is based on the premise that allogeneic MLR requires presentation of epitopes in the context of MHC class II and class I, so that suppression of responses reflects the ability of MDSC to prevent antigen-specific T-cell immune responses^27^.

Measurement of human antigen-non-specific suppression of T-cell responses by MDSC is based on the same principles as in mice, and utilizes either anti-CD3/CD28 antibody or activation with lectins. In several studies, evaluation of MDSC activity was substantiated by antibody-mediated depletion of candidate cells from PBMC before analysis of T-cell proliferation. Although this approach is less attractive, due to technical limitations in complete MDSC removal and the unintended depletion of non-MDSC that may share cross-reactive cell-surface molecules, it may provide valuable information about MDSC functional activity. To provide for better comparison between different studies, it is very important to include detailed information about the experimental protocol used for all steps involved in the purification of MDSC. Since some antibodies used for positive isolation of MDSC may modify function of these cells, it is preferable (when possible) to use negative-selection approaches. In any case, it is important to select control groups to account for possible impact of isolation techniques on MDSC function.

A number of molecules produced by MDSCs have been implicated in suppression (discussed below) including: arginases, NO, ROS, IDO, TGFβ and PGE_2_, among others. Although important for a thorough understanding of MDSC suppressive mechanism(s), evaluation of their expression cannot substitute for functional assays. In different settings MDSCs utilize different mechanisms of suppression and it is difficult to predict which will be more prevalent. It is also challenging to ascertain what level of production of any given effector molecule is sufficient for the MDSC suppressive activity.

## Biochemical and molecular characteristics of MDSCs

One of the most controversial aspects of MDSC biology is why cells with the morphology and phenotype similar to neutrophils and monocytes should require a designation of PMN-MDSC and M-MDSC. The main consideration reflects the potent immune-suppressive activity of MDSCs, the basis to define them as functionally different from monocytes and neutrophils. However, in recent years studies have identified unique biochemical and molecular characteristics of MDSC, which demonstrate that they are pathologically activated. Classical activation of neutrophils and monocytes has evolved to protect the host from bacteria and viruses, as well as to provide support for the remodelling of tissues after injury or after resolved inflammation. This activation state is characterized by robust phagocytosis, respiratory burst activity and release of pro-inflammatory cytokines. Myeloid activation is relatively short-lived and terminated on cessation of the stimulus. In contrast, pathological activation is the result of persistent stimulation of the myeloid compartment with relatively low-strength signals coming from tumours or sites of chronic inflammation. Myeloid cells generated under these conditions are poorly phagocytic, produce high levels of reactive oxygen and nitrogen species, and predominantly anti-inflammatory cytokines^9^. As a result, these cells are not able to perform effectively the normal functions of myeloid cells and acquire potent immune-suppressive potential. Reflecting their potent immunosuppressive character it possible that the main role of MDSC is in the protection of the host from extensive tissue damage caused by uncontrolled immune response associated with unresolved inflammation or infection. Tumours can hijack and amplify this activity to protect themselves from elimination by the immune system.

Since MDSC are a continuum of cells in different stages of differentiation, assignment of biochemical and molecular MDSC markers will depend on presently incompletely defined processes that govern differentiation stages of altered myelopoiesis, and some markers might thus be shared by cells with different phenotypes or with mature activated monocytes or neutrophils. For example, conversion of monocytes to granulocytes, a process known as transdifferentiation, occurs in tumour-bearing but not tumour-free mice^28^ (or at least not at the same rate). Analogously, genes contributing to the immune-suppressive phenotype, normally confined to some cell types under steady state, might be activated in MDSCs^29^.

On the basis of mostly mouse studies, four types of molecular and biochemical parameters are often associated with immune-suppressive MDSC as opposed to monocytes and neutrophils (Table 3). (1) Transcription factors and apoptosis regulators; (2) a signature of pro- and anti-inflammatory cytokines and cytokine receptors; (3) chemoattractants and related receptors affecting MDSC trafficking; and (4) enzymes and metabolic by-products contributing to MDSC immune-regulatory functions. Table 3 also indicates which molecular and biochemical parameters are often linked with the specific MDSC populations (considering PMN-MDSCs, M-MDSCs and total MDSCs). This grouping is not exhaustive, but is intended to incorporate key and non-redundant hubs related to MDSC biology. Although numbers of different parameters are implicated in MDSC biology and may help to define a molecular signature of MDSCs, some parameters are important for the development of the MDSC field and, thus, may help to identify MDSCs (highlighted in Table 3). Several assays need to be integrated to evaluate these biomarkers, which exploit gene and protein expression, as well as multiple post-translational modifications. Transcription factors may require assessment of their activity (DNA binding, phosphorylation). Technologies based on the detection of mRNA translation might be insufficient to classify MDSC as several mediators of MDSC function are regulated epigenetically or post-translationally. Epigenetic changes, such as histone modification related to myeloid differentiation, are an intensively studied and promising area of investigation, and have the potential to facilitate tracking cell stages during fate mapping analyses^30^. However, currently there is no clear indication about epigenetic markers that can discriminate specific MDSC subsets. A similar consideration is valid for microRNAs, even though in our experience microRNA signatures can be a useful classifier of myeloid cell subsets under different pathological conditions^31^.

A panoply of cytokines can either influence the properties and/or are produced by one or more subsets of MDSCs. These cytokines may establish an autocrine feed-forward loop that sustains MDSC accumulation, since they are not only released during chronic inflammation inducing MDSC, but are also released by MDSC. Chemokines and their receptors might complement the assessment of the overall inflammatory state, however, no chemokine is truly specific for MDSCs.

An immune-regulatory activity of MDSCs depends on the metabolic consumption and conversion of the amino acids L-arginine and L-tryptophan, by the activity of inducible enzymes such as arginase 1 (ARG1), nitric oxide synthase 2 (NOS2/iNOS). Since most available antibodies to these enzymes are fraught with the lack of specificity and need to access the intracellular compartment where their targets are located, detection of these enzymes is often performed by RT–PCR. However, RNA and protein levels are not necessarily coincident, and the mere presence of either RNA or protein does not confirm enzyme activation. For this reason, biochemical analyses of downstream metabolites (such as NO, kinurenines, ornithine, urea and polyamines) might be an alternative assay. MDSCs are also high producers of soluble reactive species. Reactive oxygen species exemplified by superoxide anion (O_2_^−^) and peroxide hydrogen (H_2_O_2_), are generated by the activity of NADPH oxidase (NOX) family members, in which NOX2 is likely active in myeloid cells. Reactive nitrogen species, such as the free-radical peroxynitrite (ONOO^–^), are by-products of the combined activity of iNOS, ARG1 and NOX, and can induce a number of detectable covalent alterations in select aromatic amino acids of proximal proteins by enzyme-independent nitration/nitrosylation (Table 3). The complexity of the post-translational modifications induced by peroxynitrite, the ‘nitrome', is not fully appreciated yet, but detection of nitrotyrosine and nitrotryptophan may be convenient for evaluating overall reactive nitrogen species production in biological samples, provided that more effective/specific antibodies are developed in the future. Finally, the clinical use of new checkpoint-blockade inhibitors for the therapy of human cancers, supports evaluation of their ligands (for example, PD-L1) in MDSCs, whose expression is influenced by local environmental factors such as inflammatory cytokines or hypoxia^32^,^33^.

## MDSC versus tumour-associated macrophages and neutrophils

Tumours contain a complex landscape of myeloid cells, which is associated with tumour progression and response to therapy^34^,^35^. However, the specific role of individual components of this landscape remains obscure. While the literature on tumour-associated myeloid cells assigned an unequivocal tumour-promoting activity to M-MDSCs and PMN-MDSCs^12^,^36^, both tumour-associated macrophages (TAM) and tumour-associated neutrophils (TAN) can exert either tumour-promoting or anti-tumour activities in different cancers^37^,^38^. This dual capacity stems out from the apparent phenotypic plasticity of TAM and TAN, which is modulated through their transcriptional re-programming operated by distinct micro-environmental signals, at different stages of tumour progression^37^,^38^,^39^,^40^.

**Figure 3:**
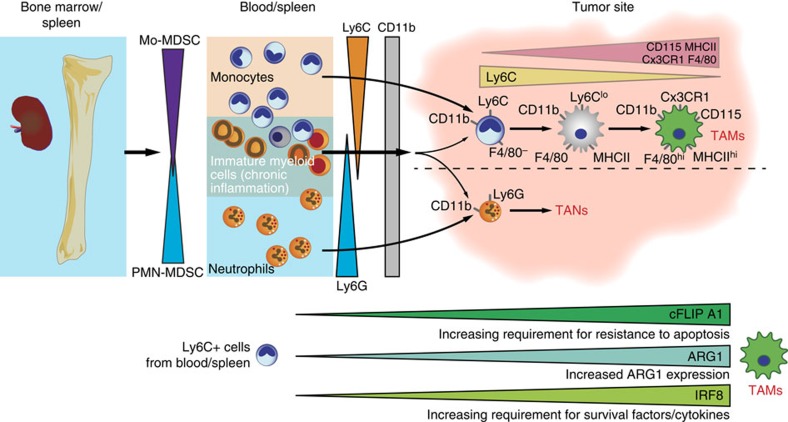
Overview of MDSC involvement in myeloid cell differentiation in cancer. In cancer and chronic inflammation, the bone marrow and spleen increase the output of mature and immature myeloid cells that comprise a spectrum between monocytes and neutrophils. In mice, MDSC toward the monocytic end of the spectrum (M-MDSC) are CD11b^+^Ly6C^+^Ly6G^−^, while towards the neutrophil end of the spectrum (PMN-MDSC) are CD11b^+^LyG^+^Ly6C^−^. Within solid tumours M-MDSC develop through intermediate steps towards macrophages where Ly6C is progressively downregulated and MHCII, F4/80 and CX3CR1 are upregulated. Under chronic inflammation, monocytic lineages show an increasing requirement for anti-apoptotic survival pathways (mediated primarily by GM-CSF signalling) to block the intrinsic mitochondrial death pathway. A similar scheme is likely to occur in humans; however, the cell markers are different.

This raises an important question of how to distinguish these cells. In mice, PMN-MDSC and TAN can be separated from mononuclear cells within the CD11b^+^ myeloid cell fraction by the expression of Ly6G granulocytic cell marker and eosinophils can be distinguished by the expression of sialic acid-binding immunoglobulin-like lectin F^41^,^42^. Mononuclear cells in tumours likely exist in various differentiation phases from monocytes/M-MDSCs towards TAM. Molecularly, this process is accompanied by the upregulation of anti-apoptotic molecules cFLIP and A1, as well as the enzyme ARG1 (refs 32, 43; Fig. 3). Phenotypically, TAM can be distinguished from M-MDSCs by increased relative expression of F4/80, low-to-intermediate expression of Ly6C and low or undetectable expression of S100A9 protein. When compared with M-MDSCs, TAM express higher amounts of IRF8, a marker of terminal macrophage differentiation, and increased M-CSF receptor, CD115 (ref. 44). Most of the published data indicate that cells with the phenotype of inflammatory monocytes (CD11b^+^Ly6C^hi^Ly6G^−^) in tumours have potent immune-suppressive activity and thus can be attributed to M-MDSCs^45^. However, whether this is indeed the case, can be clarified when specific markers of M-MDSCs are identified. There is evidence that PMN-MDSCs and M-MDSCs can be distinguished from neutrophils and monocytes due to their elevated ER stress response^46^,^47^.

In the absence of functional tests, the distinction between neutrophils and PMN-MDSCs is impossible, since these cells share the same phenotype. This limitation of phenotypical distinction between PMN-MDSCs and neutrophils only partially alleviated with additional staining with some antibodies. Compared with mature neutrophils, some PMN-MDSCs in the blood/spleen of mice have lower expression of the receptors for Fc (CD16/CD32) and complement (C5aR) and higher expression of the transcription factor retinoic-acid-related orphan receptor, an inhibitor of neutrophil maturation^37^, In some tumour models, some PMN-MDSCs, in contrast to neutrophils from naive mice, may express CD115 and CD244 (ref. 9). However, these markers have limited direct value for delineation of these cells due to apparent heterogeneity of PMN-MDSCs. A whole-transcriptomic analysis provided a pattern of gene expression that allows for discrimination between spleen PMN-MDSCs from tumour-bearing mice, and spleen and bone marrow neutrophils from tumour-free naive mice^9^,^48^.

The situation with TAN is different. TAN are a heterogeneous population of cells with some cells demonstrating pro-tumourigenic and some anti-tumour activity^38^,^49^. The distinction between TAN and PMN-MDSCs is currently impossible, since these cells share the same phenotype. A nomenclature on circulating neutrophils and TANs was recently suggested based on different parameters, such as gradient density, morphology function and tissue localization^50^. While concepts about cell subsets are certainly helpful for future discussions, mechanistic evidence about distinct maturation and differentiation steps involving neutrophils are still preliminary. For instance, the terms anti-tumourigenic N1 and pro-tumourigenic N2 mouse neutrophils were introduced to describe different populations of TAN^18^,^38^. However, the phenotype and functional characteristics of N2 neutrophils are very similar to that of PMN-MDSCs, which necessitate better characterization of these cells. This notion is supported by data demonstrating potent immune-suppressive activity by TAN, which defines these cells as PMN-MDSCs^51^,^52^,^53^. A whole-transcriptomic analysis demonstrated a substantial difference between TAN and spleen neutrophils and PMN-MDSCs^37^. However, the transcriptomic analysis of true tumour PMN-MDSCs will not be possible until markers separating these cells from TAN are discovered.

In humans, macrophage-specific markers CD68 and CD163, as well as low or absent expression of S100A9 can be used to discriminate between TAM and tumour M-MDSCs. The challenges in distinguishing between tumour PMN-MDSCs and TAN are the same as in mice: no clear cell-surface markers exist so far to allow for direct separation between TAN and PMN-MDSCs.

## Proposed experimental algorithm for MDSC reporting

**Figure 4:**
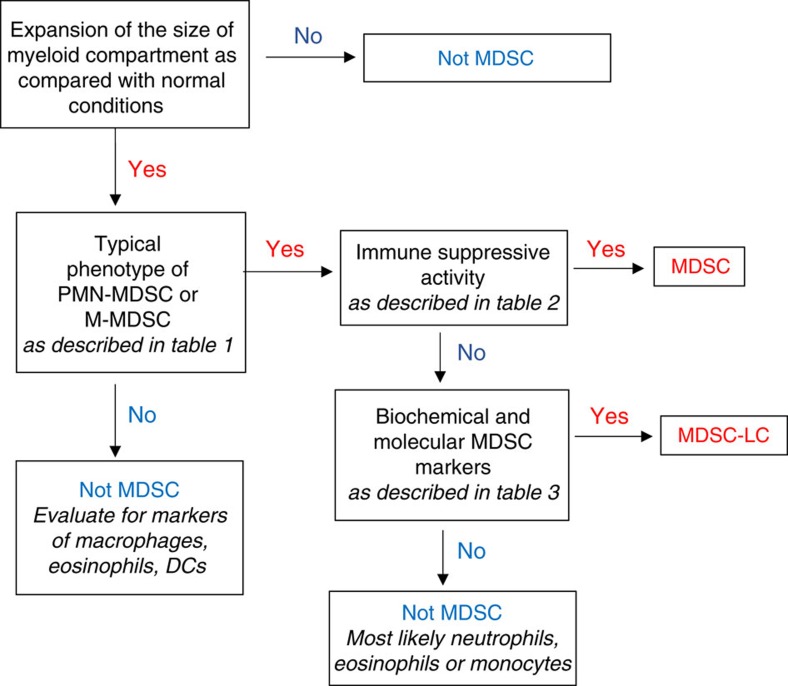
Algorithm for identification of cells as MDSC. Step-by-step approach to identify cells as MDSC for reporting. It is important, wherever possible, to use cells with the same phenotype from control mice or healthy donors as controls.

Immune-suppressive activity has been long considered as the major characteristic of MDSCs, and the lack of unique phenotypic markers made it previously impossible to characterize these cells as MDSCs if immune-suppressive activity was not detected. These cells have been referred to as TAN or monocytes^38^,^54^, but recently this designation has been refined. More sophisticated biochemical and gene-expression profiling has allowed for characterization of these cells as pathologically activated, immature myeloid cells distinct from myeloid cells found in the steady state. Immune-suppressive activity, although a critically important attribute of MDSC, is not always associated with the cells present in tumour-bearing hosts or in conditions of chronic inflammation. For example, early stages of cancer or initial stages of chronic inflammation may be associated with accumulation of cells with phenotypic (Table 1) and biochemical characteristics (Table 3) of MDSC but lacking potent suppressive activity. Moreover, it is possible that even in advanced-stage cancer, not all cells with a MDSC phenotype possess immune-suppressive activity. Although the nature of the factors responsible for acquisition of the immune-suppressive phenotype of MDSC is not entirely clear, non-suppressive MDSCs can nonetheless regulate various aspects of tumour onset and progression^5^,^12^,^36^. In fact, recent studies demonstrated that in the setting of chronic inflammation cells with an MDSC phenotype, but lacking suppressive activity contribute to the early stages of tumour inflammation^20^,^55^. Since the term ‘MDSC' presumes suppressive activity, this term should not be used to identify cells lacking this function. Therefore, when describing these cells we suggest the term MDSC-like cells (MDSC-LC; Fig. 4).

For defining MDSC populations in mice and humans, we propose an algorithmic approach that first focuses on the phenotypic characterization of the cells (Table 1). If cells meet these criteria, then suppressive activity must be determined (Table 2). The presence of suppressive activity would define these cells as MDSC. However, if cells lack suppressive activity, then major biochemical characteristics of these cells need to be evaluated (Table 3). On the basis of the available data we suggest the following criteria that could help to define MDSC-LC in the absence of suppressive activity. The analysis should include appropriate controls: cells with the same phenotype from healthy individuals or naive mice. These criteria include changes in at least two critical transcription factors and regulators—such as, IRF8, phospho-STAT3, c/EBPb, S100A8/A9, Rb1—and upregulation of at least one critical cytokine/receptor—such as, IL-10, TGFβ, IL-4R—or immune-regulatory molecule—such as,ARG1, NOS2, NOX2, PNT, PGE_2_. The number of parameters can be further expanded as new information becomes available. The absence of relevant biochemical characteristics would then exclude these cells as MDSC, whereas the presence of these markers would allow characterization of these cells as MDSC-LC (Fig. 4). Definition of molecular pathways and markers for MDSC-LC will clarify steps of the differentiation pathway induced in MDSC by growing tumours and will help to dissect tumour-promoting functions of MDSC. Furthermore, a better understanding of the developmental complexity of these cells may provide additional information on the tumour-promoting functions of MDSC that either act in a dependent or independent manner to regulate adaptive and/or innate immunity.

## Additional information

**How to cite this article:** Bronte, V. *et al.* Recommendations for myeloid-derived suppressor cell nomenclature and characterization standards. *Nat. Commun.* 7:12150 doi: 10.1038/ncomms12150 (2016).

## Figures and Tables

**Table 1:** Minimal phenotypic characteristics necessary to identify cells as MDSC.

**Mouse**	**Phenotype**	**Human (in PBMC fraction)**	**Phenotype**
Total MDSC (not sufficient for MDSC characterization)	Gr-1^+^CD11b^+^	Total (mixed) MDSC	Not clearly determined
PMN-MDSC	CD11b^+^Ly6C^lo^Ly6G^+^	PMN-MDSC	CD14^−^CD11b^+^CD15^+^(or CD66b^+^)
M-MDSC	CD11b^+^Ly6C^hi^Ly6G^−^	M-MDSC	CD11b^+^CD14^+^HLA-DR^low/−^ CD15^−^
eMDSC	Not clearly determined	e-MDSC	Lin^−^(CD3/14/15/19/56)/HLA-DR^−^/CD33^+^

eMDSC, early-stage MDSC; MDSC, myeloid-derived suppressor cell; M-MDSC, monocytic-MDSC; PBMC, peripheral blood mononuclear cell; PMN-MDSC; polymorphonuclear-MDSC.

Although phenotype is the first necessary step for defining MDSC, please note that, it cannot be used as the sole parameter for distinction between PMN-MDSC and neutrophils and M-MDSC and monocytes.

It is important, wherever possible, to use cells from control mice or healthy donors as controls.

**Table 2:** Minimal functional characteristics necessary to identify cells as MDSC.

**Mouse functional tests**		**Human functional tests**	
**Type of immune response**	**Assays**	**Autologous system**	**Allogeneic system**
· Inhibition of antigen-non-specific function (anti-CD3/CD28 or ConA induced)· Inhibition of antigen-specific function using antigen-specific T cells (induced after immunization with peptides or from transgenic mice)	· Inhibition of ^3^H-thymidine incorporation or CFSE dilution· Inhibition of CTL activity· Inhibition of IFN-γ production by T cells in ELISPOT or intracellular staining· Inhibition of expression of CD3ζ chain on T cells· Inhibition of IL-2 production	· Inhibition of anti-CD3/CD28 (or PHA) induced T-cell proliferation or IFN-γ production (in ELISPOT or by intracellular staining) by the addition of candidate MDSC populations· Improved T-cell proliferation after removal of candidate MDSC populations	· Inhibition of proliferation or IFN-γ production by T cells (in ELISA, ELISPOT or by intracellular staining) by the addition of selected MDSC populations

CTL, cytotoxic T lymphocyte; ELISA, enzyme-linked immunosorbent assay; IFN, interferon; IL, interleukin; MDSC, myeloid-derived suppressor cell.

It is important, wherever possible, to use cells from control mice or healthy donors as controls.

For either antigen-specific or antigen-non-specific response, one assay is usually sufficient.

**Table 3:** Biochemical and molecular parameters associated with MDSC characterization.

**Class of biomarkers**	**Biomarker**	**Detection technology**	**Mainly found in**	**Reference**
Transcription factors and apoptotic regulators	↓ IRF8* Phospho-STAT3*cEBP/β*S100A8/9*RBPhospho-STAT5ROR/RORC1sXBP, CHOP	FC, PPTM (FC, ELI), FAELI, P, T, FAELI, FC, IHC, P, TIF, P, T, FCPTM (FC, IHC, P), FA FC, PP, T	MDSCMDSCMDSCMDSCM-MDSC>PMN-MDSCMDSCPMN-MDSCMDSC	56,57,58,59,26,31,60,61,28†^62^,^44^,^46^,^47^
Genes and molecules contributing to the immune-regulatory activity	ARG1*NOS2/NO*NOX2/ROS*PNT/RNS*VEGFPGE_2_PD-L1	E, FC, IHC, P, TFC, IF, IHC, P, TE, FC, P, TPTM (IHC), E, FCFC, IHCELIFC, P, T	M-MDSC M-MDSCPMN-MDSCMDSCMDSCM-MDSCMDSC	63,64,65,64,8,66,46,59,67,27,68,65,69,70,71,72,73
Cytokines and receptors	IL-10*TGFβ*IL-4R (CD124)*	ELI, FC, TELI, FC, T, PFC, T	MDSCM-MDSCM-MDSC	74,75,66,76,63,77
*Cytokines involved in MDSC development*				
These cytokines are not produced by MDSC. However, they are important for the evaluation of MDSC microenvironment.	GM-CSFG-CSFIL-13IL-1	ELI, TELI, TFCELI	MDSCPMN-MDSCM-MDSCMDSC	21,78,79,21,79,80,63,81,22,74,75

E, enzyme assay; ELI, enzyme-linked immunosorbent assay; FA, functional activity (for example, DNA binding); FC, flow cytometry (including ICS, intracellular staining); IF, immunofluorescence; IHC, immunohistochemistry; IL, interleukin; MDSC, myeloid-derived suppressor cell; M-MDSC, monocytic-MDSC; P, protein detection in cell extracts or supernatants (that is, by western blot, mass spectrometry); PMN-MDSC; polymorphonuclear-MDSC; PTM, post-translational modification; T, transcript analysis (that is, by RT–PCR, RNA-seq or *in situ* hydridization).

It is important, wherever possible, to use cells from control mice or healthy donors as controls.

PMN-MDSCs are compared with neutrophils and M-MDSCs are compared with monocytes.

Total population of MDSCs in most of these studies was compared with Gr-1^+^CD11b^+^ cells from control mice. Characteristics described in the table are the same for MDSC and MDSC-LC.

^*^Parameters crucial for MDSC biology and, thus, for their identification.

^†^In this study Rb expression was largely compared between the groups of M-MDSC and PMN-MDSC.
